# A rare polymorphism in *Toll Like Receptor 2* is associated with systemic sclerosis phenotype and increases production of inflammatory mediators

**DOI:** 10.1186/1479-5876-9-S2-O4

**Published:** 2011-11-23

**Authors:** Jasper CA Broen, Lara Bossini-Castillo, Lenny van Bon, Madelon C Vonk, Hanneke Knaapen, Lorenzo Beretta, Blanca Rueda, Roger Hesselstrand, Ariane Herrick, Jane Worthington, Nicholas Hunzelmann, Christopher Denton, Carmen Fonseca, Gabriela Riemekasten, Hans Kiener, Raffaella Scorza, Carmen P Simeon, Norberto Ortego-Centeno, Miguel A Gonzalez-Gay, Paolo Airo’, Marieke JH Coenen, Javier Martin, Timothy RDJ Radstake

**Affiliations:** 1 Dept. of Rheumatology, Radboud University Nijmegen Medical Center, Nijmegen, The Netherlands; 2 Instituto de Parasitología y Biomedicina, CSIC, Granada, Spain; 3 Referral Center for Systemic Autoimmune Diseases, Fondazione IRCCS Ca’ Granda Ospedale Maggiore Policlinico and University of Milan, Italy; 4 Dept. of Rheumatology, Lund University, Lund, Sweden; 5 Rheumatic Diseases Centre, University of Manchester, Salford Royal NHS Foundation Trust, UK; 6 Dept. of Dermatology, University of Cologne, Germany; 7 Centre for Rheumatology, Royal Free and University College Medical School, London, UK; 8 Dept. of Rheumatology and Clinical Immunology, Charité University Hospital and German Rheumatism Research Centre, a Leibniz institute, Berlin, Germany; 9 Dept. of Internal Medicine, Division of Rheumatology, University of Vienna, Austria; 10 Servicio de Medicina Interna, Hospital Valle de Hebron, Barcelona, Spain; 11 For the Spanish Systemic Sclerosis group; Servicio de Medicina Interna, Hospital Universitario Central de Asturias, Oviedo, Spain; 12 Servicio de Medicina Interna, Hospital Clinico Universitario, Granada, Spain; 13 Servizio di Reumatologia ed Immunologia Clinica, Spedali Civili, Brescia, Italy; 14 Dept. of Human Genetics, Radboud University Nijmegen Medical Center, Nijmegen, The Netherlands

## Background

Toll like receptors play an important role in fine-tuning innate immune responses, but genetic variations in *TLR* genes have been shown previously to augment immune responses and susceptibility to autoimmune disease.

## Aim

To investigate whether polymorphisms in *toll like receptor* (*TLR*) genes, previously reported to be associated with immune mediated diseases are implicated in systemic sclerosis (SSc).

## Methods

We genotyped 14 polymorphisms in the *TLR 2*, *4*, *7*, *8* and *9* genes in a discovery cohort comprising 452 SSc patients and 537 controls and a replication cohort consisting of 1170 SSc patients and 925 controls. Furthermore we analyzed 15 year follow-up data from 964 patients to assess the potential association of *TLR* variants with the development of disease complications. Next to this, we analyzed the functional impact of the associated polymorphism on monocyte derived and myeloid dendritic cells.

## Results

Exploiting the discovery cohort, we observed that a rare functional polymorphism in *TLR2* (Pro631His), was associated with anti-topoisomerase positivity (p=0.003 OR 2.24 95%CI:1.24-4.04). This observation was validated in the replication cohort (p=0.0001 OR 2.73 95%CI:1.85-4.04). In addition, the replication cohort also revealed an association between the *TLR2* variant with the diffuse subform of the disease and the development of pulmonary arterial hypertension, respectively (p=0.02, Log-Rank p=0.003, Cox proportional hazards ratio: 5.61 ((95%CI 1.53-20.58)). Functional analysis revealed that monocyte derived dendritic cells carrying the Pro63His variant produce more inflammatory mediators (TNFalpha and IL-6) upon TLR2 mediated stimulation (both p<0.0001).

## Conclusion

The rare TLR2Pro631His variant is robustly associated with anti-topoisomerase positivity, diffuse SSc and the development of PAH. Besides, this variant influences TLR2 mediated cell responses. Further research is necessary to reveal the precise role of TLR2 in the disease pathogenesis of SSc.

